# Complete, Closed Genome Sequences of 10 *Salmonella enterica* subsp. *enterica* Serovar Typhimurium Strains Isolated from Human and Bovine Sources

**DOI:** 10.1128/genomeA.01212-16

**Published:** 2016-11-03

**Authors:** Scott V. Nguyen, Dayna M. Harhay, James L. Bono, Timothy P. L. Smith, Patricia I. Fields, Blake A. Dinsmore, Monica Santovenia, Christy M. Kelley, Rong Wang, Joseph M. Bosilevac, Gregory P. Harhay

**Affiliations:** a USDA-ARS, U.S. Meat Animal Research Center, Clay Center, Nebraska, USA; b Enteric Diseases Laboratory Branch, Division of Foodborne, Waterborne, and Environmental Diseases, Centers for Disease Control and Prevention, Atlanta, Georgia, USA

## Abstract

*Salmonella enterica* is a leading cause of enterocolitis for humans and animals. *S. enterica* subsp. *enterica* serovar Typhimurium infects a broad range of hosts. To facilitate genomic comparisons among isolates from different sources, we present the complete genome sequences of 10 *S*. Typhimurium strains, 5 each isolated from human and bovine sources.

## GENOME ANNOUNCEMENT

With a global burden of ~94 million cases each year, *Salmonella* infections diminish human health, well-being, and productivity, while negatively impacting the global economy, and present an ongoing public health challenge (1). *S. enterica* subsp. *enterica* serovar Typhimurium (*S*. Typhimurium) is one of the most common serovars encountered in clinical settings, notably with the global expansion of a multidrug-resistant DT104 phage-type clone in the last few decades (2). *S*. Typhimurium can be isolated from a broad range of sources, including produce, dairy, pork, poultry, and beef (3). As many of the virulence factors and antimicrobial resistance (AMR) genes in *S*. Typhimurium are carried on mobile genetic elements and plasmids, complete genome sequence data allow the construction of improved phylogenetic trees that may be used to characterize the distribution and evolution of these elements. Furthermore, comparative genomic analyses increase our understanding of the genetic diversity of these important human and livestock pathogens. To facilitate these analyses, we present the complete, closed genome and plasmid sequences for 10 *S*. Typhimurium strains isolated from bovine sources (ground beef, cattle hides, and pre-evisceration beef carcasses) and human clinical cases of salmonellosis.

Genomic DNA was purified with Qiagen Genomic-tip 100/G columns and Blood & Cell Culture DNA Midi kits (Qiagen, USA), using the manufacturer’s recommended protocol, from overnight cultures grown at 37°C in trypticase soy broth (Becton, Dickinson, USA). Single-molecule real-time sequencing libraries of bacterial DNA were constructed as per the manufacturer’s protocol using P4-C2 or P5-C3 chemistry and sequenced using a PacBio RS II instrument (Pacific Biosciences, USA), producing average subreads of >7 kb and mean genome coverage of 134×. Genomes were assembled using Celera version 7.0 (4) and then validated and checked by Quiver (5). Geneious version 9.0.5 (Biomatters Ltd., New Zealand) (6) was used to trim the sequences of duplicate ends. OriFinder (7) was used to determine the origin of replication, and the origin was set to nucleotide position 1 for depositing into NCBI. Genome and plasmid sequence data were annotated using the NCBI Prokaryotic Genome Annotation Pipeline and deposited into NCBI GenBank. Noteworthy in the assembly of CDC 2010K-1587 was a 208.9 kb contig representing a putative large plasmid. However, *in silico* replicon (8) typing revealed the presence of two different replicons, suggesting that the large contig was actually composed of two smaller plasmids. Gel electrophoresis (9) and PCR amplification of targeted plasmid sequences confirmed the presence of two plasmids at 104.6 (pSTY1-2010K-1587; IncA/C) and 104.2 kb (pSTY2-2010K-1587; IncI), but additionally confirmed the faint presence of the 208.9-kb hybrid plasmid.

### Accession number(s).

Nucleotide accession numbers, sizes (bp), and phenotypic AMR phenotypes of the strains are listed in Table 1.

**TABLE 1:** Chromosome and plasmid sequence accession numbers and additional information for 10 *Salmonella enterica* subsp. *enterica* serovar Typhimurium strains

Strain or plasmid	NCBI accession no.	Size (bp)	AMR phenotype^a^	Source of isolation
pSTY1-1899^b^	CP014962	93,850	PS	
USMARC-1808	CP014969	4,936,898	AmApCSSuTe	Bovine post-evisc
pSTY1-1808	CP014970	94,014		
USMARC-1810	CP014982	4,927,737	ApKSSu	Bovine pre-evisc
USMARC-1880	CP014981	4,815,205	PS	Bovine pre-evisc
USMARC-1896	CP014977	4,856,402	AmApFTAxCSSuTe	Bovine fat trim
pSTY1-1896	CP014978	147,296		
USMARC-1898	CP014971	4,809,521	PS	Ground beef
pSTY1-1898	CP014972	95,774		
pSTY2-1898	CP014973	93,960		
pSTY3-1898	CP014974	35,954		
CDC 2009K-1640	CP014975	4,933,708	(Am)ApCSSuTe	Human stool
pSTY1-2009K-1640	CP014976	94,019		
CDC 2009K-2059	CP014983	4,823,797	PS	Human stool
CDC 2010K-1587	CP014965	4,799,415	AmApFTAxKSuTe	Human stool
pSTY1-2010K-1587	CP016864	104,649		
pSTY2-2010K-1587	CP016865	104,250		
pSTY3-2010K-1587	CP016866	4,675		
pSTY4-2010K-1587	CP016867	3,223		
CDC 2011K-1702	CP014967	4,906,324	(Am)ApSu	Human urine
pSTY1-2011K-1702	CP014968	94,016		
CDC H2662	CP014979	4,891,165	(Am)ApCSSuTe	Human stool
pSTY1-H2662	CP014980	94,034		

a Antimicrobial resistance (AMR) determined by broth microdilution (CMV2AGNF, Sensititre, Trek Diagnostics, Thermo, Fisher) using CLSI minimum inhibitory concentration (MIC) breakpoints. AMR phenotype key: PS, pan-susceptible; Am, amoxicillin-clavulanic acid; Ap, ampicillin; F, cefoxitin; T, ceftiofur; Ax, ceftriaxone; C, chloramphenicol; K, kanamycin; S, streptomycin; Su, sulfisoxazole; Sxt, sulfamethoxazole-trimethoprim; Te, tetracycline; (Am), amoxicillin-clavulanic acid (MIC = 16) intermediately resistant; bovine pre-evisc, pre-evisceration carcass; bovine post-evisc, post intervention carcass.

b Host strain USMARC-1899 submitted to NCBI GenBank previously (10).
